# Infection prevention and control (IPC) implementation in low-resource settings: a qualitative analysis

**DOI:** 10.1186/s13756-021-00962-3

**Published:** 2021-07-31

**Authors:** Sara Tomczyk, Julie Storr, Claire Kilpatrick, Benedetta Allegranzi

**Affiliations:** 1 Infection Prevention and Control Technical and Clinical Hub, Department of Integrated Health Services, World Health Organization, Geneva, Switzerland; 2 Institute of Global Health, University of Geneva, Geneva, Switzerland

**Keywords:** Infection prevention and control, Implementation, Low-resource settings, Qualitative evidence, WHO core components

## Abstract

**Background:**

The coronavirus disease-2019 (COVID-19) pandemic has again demonstrated the critical role of effective infection prevention and control (IPC) implementation to combat infectious disease threats. Standards such as the World Health Organization (WHO) IPC minimum requirements offer a basis, but robust evidence on effective IPC implementation strategies in low-resource settings remains limited. We aimed to qualitatively assess IPC implementation themes in these settings.

**Methods:**

Semi-structured interviews were conducted with IPC experts from low-resource settings, guided by a standardised questionnaire. Applying a qualitative inductive thematic analysis, IPC implementation examples from interview transcripts were coded, collated into sub-themes, grouped again into broad themes, and finally reviewed to ensure validity. Sub-themes appearing ≥ 3 times in data were highlighted as frequent IPC implementation themes and all findings were summarised descriptively.

**Results:**

Interviews were conducted with IPC experts from 29 countries in six WHO regions. Frequent IPC implementation themes including the related critical actions to achieve the WHO IPC core components included: (1) To develop IPC programmes: continuous advocacy with leadership, initial external technical assistance, stepwise approach to build resources, use of catalysts, linkages with other programmes, role of national IPC associations and normative legal actions; (2) To develop guidelines: early planning for their operationalization, initial external technical assistance and local guideline adaption; (3) To establish training: attention to methods, fostering local leadership, and sustainable health system linkages such as developing an IPC career path; (4) To establish health care-associated (HAI) surveillance: feasible but high-impact pilots, multidisciplinary collaboration, mentorship, careful consideration of definitions and data quality, and “data for action”; (5) To implement multimodal strategies: clear communication to explain multimodal strategies, attention to certain elements, and feasible but high-impact pilots; (6) To develop monitoring, audit and feedback: feasible but high-impact pilots, attention to methods such as positive (not punitive) incentives and “data for action”; (7) To improve staffing and bed occupancy: participation of national actors to set standards and attention to methods such as use of data; and (8) To promote built environment: involvement of IPC professionals in facility construction, attention to multimodal strategy elements, and long-term advocacy.

**Conclusions:**

These IPC implementation themes offer important qualitative evidence for IPC professionals to consider.

**Supplementary Information:**

The online version contains supplementary material available at 10.1186/s13756-021-00962-3.

## Background

Health care-associated infections (HAIs) represent a major burden for health care system delivery and patient safety. However, evidence has clearly demonstrated that effective implementation of evidence-based infection prevention and control (IPC) interventions can lead to substantial reductions in HAIs [1]. The emergence of the coronavirus disease-2019 (COVID-19) has again shown the importance of effective IPC implementation to prevent and control infectious disease outbreaks in health care. Nosocomial transmission of Severe acute respiratory syndrome coronavirus 2 (SARS-CoV-2) has been observed globally, and it has revealed the need for renewed and continued focus on building effective IPC programmes, particularly in low-resource settings [2–7].

In a large multi-country study, national IPC focal points were interviewed to assess the level of implementation of IPC programmes at the national level [8]. Among the low-income countries interviewed, only nine (45%) reported the existence of a national IPC programme; four (20%) had documents on implementation strategies; one (5%) monitored compliance with IPC practices. Experts have stressed that standards such as the World Health Organization (WHO) IPC programme minimum requirements need to be in place in health care in order to effectively manage infectious disease threats, particularly in low-resource settings where the burden of HAIs has been shown to be higher [9]. Notably, the pooled prevalence of HAIs in resource-limited settings was found to be 15.5 per 100 patients among high quality studies compared to the average prevalence of HAIs in Europe (7.1 per 100 patients) and the estimated incidence in the USA (4.5 per 100 patient) in a 2011 systematic review [10]. In regions such as the African continent, progress has been made in the area of outbreak preparedness and IPC since the 2014–2016 Ebola outbreak [11]. However, improved evidence and sufficient resources are critical to enabling the long-term effectiveness and sustainability of these IPC practices.

Robust evidence on effective IPC implementation strategies in low-resource settings remains limited. In an effort to add to the body of evidence, we aimed to qualitatively assess examples of IPC implementation in these settings and summarize key learned lessons.

## Methods

A standardized questionnaire assessing IPC implementation experiences was developed based on the WHO core components of IPC programmes and first piloted to provide valid framework [1]. Using the questionnaire, two interviewers with IPC experience conducted a series of semi-structured interviews with IPC professionals from low-resource settings in 2018. A convenience sample of interviewees was asked to participate based on the following criteria: (1) long-term work experience in leadership roles in a limited resource setting and (2) demonstrated IPC expert competencies such as certification and past collaboration with the WHO IPC Technical and Clinical Hub. Attempts were also made to include experts from a broad range of geographical regions, although interviews were conducted in English.

A qualitative inductive thematic analysis was used by one analyst to assess the results of the semi-structured interviews and identify patterns within the data [12]. In the first phase, initial codes were generated for IPC implementation examples that appeared meaningful in interview transcripts using a semantic and realist approach. In the second phase, codes were collated into sub-themes. These were defined as patterned elements across data items which captured key IPC implementation learned lessons. The sub-themes were then grouped again into broad overarching themes. In the third phase, all themes were reviewed and refined to ensure that they were valid and adequately captured the data, also in the context of previous IPC evidence. The themes identified by the analyst were also discussed and validated with two additional IPC experts.

The final (sub-)themes were summarized descriptively. Based on the relative distribution of findings according to theme, sub-themes appearing ≥ 3 times in data items were defined as “frequent” IPC implementation lessons learned for each of the WHO IPC core components, providing insights on successful approaches for improvement and critical areas for attention [13]. Those occurring < 3 times were reported as other “unique” implementation ideas. Selected transcript quotes were used to illustrate some key learned lessons. The statistical software R (version 3.6.2) was used for all analyses. The interviews and analysis were conducted as part of a routine evaluation activity of the WHO IPC Technical and Clinical Hub.

## Results

Twenty-nine interviews were conducted with the selected IPC experts. Interviewees included fourteen (48.3%) from the African region (Botswana, Burkina Faso, Congo, Ethiopia, Ghana, Guinea, Kenya, Liberia, Malawi, Nigeria, Senegal, Sierra Leone, South Africa, and Zimbabwe), five (17.2%) from the Pan-American region (Argentina, Barbados, Brazil, Chile, Jamaica), three (10.3%) from the European region (Baltic states, Georgia, Hungry), two (6.9%) from the Eastern Mediterranean region (Egypt, Pakistan), two (6.9%) from the Western Pacific region (India, Vietnam) and one (3.4%) from the South-East Asian region (Sri Lanka) as well as two (6.9%) from international organizations active in providing IPC in a range of limited-resource settings (Médecins San Frontières and WHO Water Sanitation and Hygiene (WASH) Department).

**Fig. 1:**
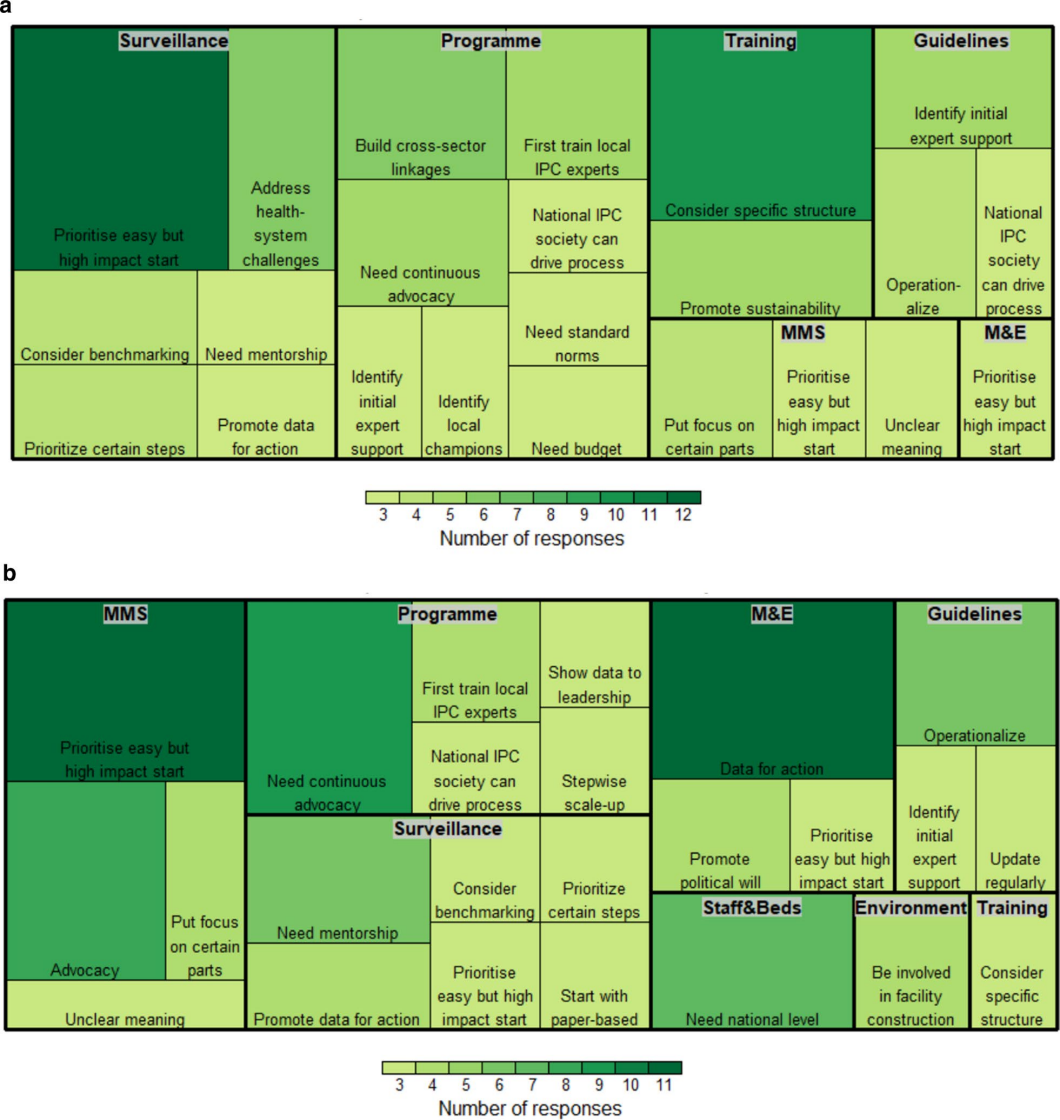
**a** IPC implementation themes according to each WHO core component at the national level. *Abbreviations: WHO IPC Core Components including 1) Programme: IPC programme; 2) Guidelines: IPC guidelines; 3) Training: IPC education and training; 4) Surveillance: HAI surveillance; 5) MMS: Multidmodal strategies for implementation of IPC interventions; 6) M&E: Monitoring/audit of IPC practices and feedback; 7) Staff&Beds: Workload, staffing and bed occupancy; and 8) Environment: Built environment, materials and equipment for IPC. **b** IPC implementation themes according to each WHO core component at the acute health care facility level

The qualitative analysis revealed a range of frequent (≥ 3 occurrences) themes of lessons learned reported by IPC experts (Table 1), as well as other unique implementation ideas (< 3 occurrences; Additional file 1). The themes could also be distinguished by those reported for activities at the national level (Fig. 1a) or at the acute health care facility level (Fig. 1b).

**Table 1:** Frequent (≥ 3 occurrences) themes concerning IPC implementation lessons learned in low-resource settings according to each WHO core component

Theme	Sub-themes	N*
*Core Component 1: IPC programme*		
Need an approach to maintain “continuous” advocacy (n = 15)	Set up regular meetings with senior leadership/managers	10
	IPC should be a part of routine meetings, presentations, or rounds	5
May first need external technical assistance (n = 13)	National level should first support selected professionals to receive external IPC training and these professionals can then act as trainers in-country	9
	External IPC experts should first review initial materials to ensure they meet IPC standards	4
Use a stepwise approach to build required resources (n = 10)	Start with a small group of committed staff in addition to link nurses and regional staff	6
	Need at least a small budget in the beginning for recognition	4
Use specific activities or opportunities as “catalysts” for advocacy (n = 6)	Use of data (process or outcome measures) can help convince leadership of IPC’s importance, i.e. avoid "no data, no problem"	3
	Publicize starting examples, e.g. hand hygiene, surgical site infections	3
Promote linkages with health system (n = 6)	Link IPC personnel and team with the quality management team	3
	Link IPC personnel and team with AMR team	3
National IPC association can drive IPC improvement (n = 6)	National IPC association can be active in providing expert input and assisting with local adaption of materials	6
May need normative actions to convince stakeholders (n = 4)	Need legislation for recognition	4
*Core Component 2: IPC guidelines*		
Consider specific approaches to operationalize guidelines (n = 16)	Link guidelines directly to training and workshops	6
	Link guidelines directly to monitoring indicators	4
	Set guideline dissemination plan early during planning	3
	Designate dedicated multidisciplinary guideline implementation leads	3
Use specific strategies for adaption of guidelines (n = 15)	Schedule ongoing meetings to review guidelines and regularly update them based on current evidence and practice	5
	National IPC association can drive guidance development and adaption	4
	Meet with other public health programmes (e.g. maternal and child health, HIV, tuberculosis) and identify joint guideline themes and actions	3
	Develop a plan to collect local evidence to inform guidelines	3
May first need external technical assistance (n = 12)	Hire external IPC expert for initial development and then locally adapt	8
	Adapt international standard guidelines, e.g. WHO, ECDC, US CDC	4
*Core Component 3: IPC education and training*		
Consider specific training methods (n = 19)	Select 1–2 master trainers to first receive IPC expert training outside of the country	5
	Consider multidisciplinary training, i.e. different staff together, to remove hierarchy	4
	Use a train-the-trainers structure	4
	May need initial IPC expert technical consultant and then can locally adapt training	3
	Ensure regular in-service workshops	3
Promote linkages with health system and sustainability (n = 9)	Create an IPC career path, e.g. accreditation	5
	Harmonize trainings across programmes, e.g. maternal and child health, HIV, tuberculosis	4
Foster local IPC leadership during trainings (n = 7)	Require mandatory trained IPC hospital leads who can play an integral role in trainings	4
	Identify local champion trainers and trainees at the facility level	3
*Core Component 4: HAI surveillance*		
Prioritise feasible but high-impact starting points or pilots (n = 30)	Start with surgical site infection (e.g. post caesarean-section, 30-day follow-up) pilot	8
	Start with device-associated infection, e.g. urinary or bloodstream, pilot	5
	Start with severe acute respiratory infection pilot	5
	Use a stepwise fashion to slowly scale-up surveillance in a careful way	5
	Can start with paper-based system but develop transition plan for electronic surveillance	4
	Start with pilot in intensive care units	3
Ensure multidisciplinary collaboration, mentorship (n = 26)	Conduct regular surveillance training and feedback, e.g. yearly seminars	6
	Conduct site support visits, e.g. assessment of case finding, forms, denominator data	5
	Advocate for integration of HAI surveillance with AMR and stewardship efforts	4
	Create a technical working group on surveillance in National IPC or AMR committees	4
	Ensure that one hospital is effectively trained in surveillance and can provide leadership to other hospitals	4
	Promote frequent informal mentorship	3
Carefully consider definitions and data quality processes (n = 22)	Conduct a careful structured discussion on adaption of case definitions, maintaining standards, consistency and predictive value	7
	Reference US National Healthcare Safety Network (NHSN) definitions	7
	First identify who can collect, clean, and analyse data, i.e. invest in statisticians	4
	Decide early on how to regularly evaluate data quality	4
Promote “data for action” (n = 7)	Leverage quality improvement programme/activities	7
*Core Component 5: Multidmodal strategies for implementation of IPC interventions*		
Promote activities to clearly communicate and advocate for multimodal strategies (n = 16)	Need leadership buy-in to obtain resources, e.g. awareness workshop, regular meetings	7
	Many cannot explain what multimodal strategies so communicate a clear definition	6
	Identify multidisciplinary champions for multimodal strategies	3
Put focus on certain elements of multimodal strategies (n = 16)	Monitoring, audit, feedback, scoring and accountability mechanisms are key elements	8
	Guidelines and training are key elements	4
	Promotion of safety culture is a key element, e.g. organizational culture questionnaire, team communication mechanisms, mentorship activities	4
Prioritise feasible but high-impact starting points or pilots (n = 14)	Start with hand hygiene pilot	8
	Start with device-associated infections, e.g. urinary or bloodstream, pilot	3
	Start with surgical site infection pilot	3
*Core Component 6: Monitoring/audit of IPC practices and feedback*		
Promote “data for action” (n = 17)	Present at IPC committee meetings, during hospital workshops, and in staff emails to build political will for change	6
	Recognize performance with incentives, e.g. centre of excellence, ward/personnel awards	6
	Publish scores for staff, e.g. device-associated infection-free days, hand hygiene practices	5
Prioritise feasible but high-impact starting points or pilots (n = 12)	Monitoring/audit and feedback should be part of IPC implementation from the beginning	5
	Start with hand hygiene pilot	4
	Start small to show “the problem”	3
Put focus on certain methods (n = 6)	Communicate positive audit and feedback culture, i.e. not punitive	3
	Integrate with national health monitoring and information systems (HMIS)	3
*Core Component 7: Workload, staffing and bed occupancy*		
Need the participation of national level actors (n = 11)	National level actors should set standards, e.g. for nurse-patient ratio	6
	Long-term advocacy with national level actors is essential	5
Put focus on certain methods (n = 3)	Need to show data and local research to set staffing and bed occupancy standards	3
*Core Component 8: Built environment, materials and equipment for IPC*		
IPC professionals should be actively involved in facility construction (n = 8)	Conduct regular meetings between construction and IPC teams to ensure that facility design, construction, modifications and renovations meet IPC standards	8
Put focus on certain elements of a multimodal strategy (n = 5)	Start with procuring equipment for hand hygiene	5
Promote long-term advocacy and integration with health system (n = 3)	Long-term WASH advocacy is needed for leadership buy-in and need phased in approach	3

*Themes and sub-themes are listed in order of decreasing frequency for each WHO core component of IPC programmes

**AMR (Antimicrobial Resistance), ECDC (European Centre for Disease Prevention and Control), HAI (Health Care-Associated Infections), HIV (Human Immunodeficiency Virus), IPC (Infection Prevention and Control), NHSN (National Healthcare Safety Network), US CDC (United States Centers for Disease Control and Prevention), WASH (Water Sanitation and Hygiene), WHO (World Health Organization)

### IPC programmes

The first WHO IPC core component recommends the establishment of an IPC programme at the national and facility level. Seven frequent themes were identified as important for the implementation of this component (Table 1): an approach for maintaining continuous advocacy such as inclusion of IPC in routine meetings and regular meetings with leadership (n = 15); initial external technical assistance such as training outside of the country for selected local professionals or the review of initial protocols by external experts (n = 13); and a stepwise approach to build required resources such as starting with a small budget and a few committed staff (n = 10). In addition, the use of specific activities as catalysts such as feedback on process or outcome measures or publication of IPC examples (n = 6), the promotion of linkages such as with quality management, antimicrobial resistance (AMR) or the nursing directorate (n = 6), the role of the national IPC association for driving IPC improvement (n = 6) and normative or legal actions to convince stakeholders (n = 4) were also noted. Other unique ideas included the use of regular emails to leadership, research grant programmes, outbreaks, patient associations, and citing international norms to build political will as well as first asking the administration to dedicate one to two working days to IPC (Additional file 1). The following statements by interviewees further illustrate the lessons learned for the first and critical core component:

*“We first needed baseline data to convince leadership measures to break the cycle of ‘no data, no problem’ so we started with monitoring, audit and feedback of hand hygiene as our first activity to build our IPC programme. We spent a long time regularly discussing the results and needs in meetings and copying leadership on all important IPC emails. Eventually, we received a small budget line item for IPC.”*

*“At the beginning, it was difficult to gain support for IPC. We had to find a way to build a programme step-by-step over time. I convinced the hospital leadership to first support by my external IPC training outside of the country to get certified. I identified three committed nurses and leadership agreed to have them work 1-2 days per week only for IPC.”*

*“The key challenge with IPC programme development is political will. In our region, legislation is very important. The countries need to have a norm talking about the obligation of an IPC committee. Then, one can work advocacy to show IPC as a value rather than an obstacle.”*

*“We first got some small amount of funding to do a research project on surgical site infections. Then we held regular patient safety rounds with senior executives and clinicians and discussed the importance of IPC whenever we could. Then we started a facility IPC committee and work on installing alcohol-based hand rub stations at the point of care and staff education.”*

### IPC guidelines

The second WHO IPC core component recommends the development of evidence-based IPC guidelines and related activities to train health workers on the recommended practices. Three frequent themes were identified as important for the implementation of this component (Table 1): early planning for operationalizing guidelines such as linking guidelines to training, monitoring and implementation leads (n = 16); strategies for the adaption of guidelines such as the adaption of international standards with local evidence, regular updates based on current evidence, linkages to other public health programmes and the involvement of the national IPC association (n = 15); and initial external technical assistance (n = 12). Other unique ideas included linking guidelines to standard operating procedures, pocket “how-to” guides, research or smartphone applications as well as setting guideline implementation deadlines (Additional file 1).

### IPC education and training

The third WHO IPC core component recommends the establishment of IPC education and training at the facility level with national level support. Three frequent themes were identified as important for the implementation of this component (Table 1): the use of specific training methods such as selected master trainers to first receive training outside of the country, multidisciplinary trainings to remove staff hierarchy, and regular in-service workshops (n = 19); the promotion of sustainable linkages with the health system such as the creation of an IPC career path and the harmonization of trainings across other public health programmes (n = 9); and fostering local IPC leadership during trainings such as the identification of champion hospital trainers and trainees (n = 7). Other unique ideas included integrating training competencies directly into job descriptions and performance reviews, requiring participants to pay a small fee to sustain the programme, conducting training also for administrators, the use of ongoing mentorship, inclusion of communication skills in training content, inclusion of IPC in new employee orientation, regional meetings to keep experts updated and collaboration with other Ministries, universities or associations (Additional file 1).

### HAI surveillance

The fourth WHO IPC core component recommends the establishment and performance of HAI surveillance. Four frequent themes were identified as important for the implementation of this component (Table 1): the prioritisation of feasible but high-impact pilots such as starting with surgical site infection or device-associated infection in intensive care units and slowly scaling-up in a stepwise manner and working from paper to electronic forms (n = 30); ensuring multidisciplinary collaboration and mentorship such as yearly hospital surveillance seminars, site support visits to assess case finding and denominator data, the integration of HAI and AMR surveillance efforts including a multidisciplinary working group and a master hospital trained in surveillance that could provide leadership to others (n = 26); careful consideration of definitions such as National Healthcare Safety Network (NHSN) before use (i.e. need to discuss factors such as the use of validated standards, consistency and feasibility of data collection) as well as clear procedures for data management (n = 22); and promotion of “data for action” leveraging quality improvement activities (n = 7). Other unique ideas included starting with process indicators before outcome surveillance, starting with a neonatal sepsis pilot, surgical site infection surveillance, clinical surveillance with round observations and chart reviews, surveillance as part of a research project, or using tuberculosis data to show “the problem”, sending monthly reports, displaying HAI rates on ward notice boards, discussing data in rounds, using International Nosocomial Infection Control Consortium (INICC) [14] for benchmarking, advocating for harmonized definitions and methods across states, and collaborating with the Information Technology (IT) department (Additional file 1).

### Multimodal strategies

The fifth WHO IPC core component recommends the use of multimodal strategies for the implementation of IPC interventions. Three frequent themes were identified as important for the implementation of this component (Table 1): the promotion of activities to clearly communicate and advocate for multimodal strategies such as encouraging leadership buy-in to obtain resources, teaching others the meaning of multimodal strategies and the identification of champions (n = 16), putting focus on certain elements of the multimodal strategies such as monitoring, audit and feedback, guidelines and training and promotion of safety culture (n = 16), and the prioritisation of feasible but high-impact pilots such as strategies addressing hand hygiene, device-associated infections or surgical site infections (n = 14). Other unique ideas included emphasizing the local production of alcohol-based hand rub and environmental cleaning as key elements, integration of bundle interventions and quality checklists, establishing facility goals and standards for multimodal strategies, and the adaption of successful multimodal strategy examples from other countries (Additional file 1).

### Monitoring, audit and feedback

The sixth WHO IPC core component recommends the implementation of monitoring or audit of IPC practices and feedback. Three frequent themes were identified as important for the implementation of this component (Table 1): the promotion of “data for action” such as presenting data at meetings or in staff emails, recognizing performance with incentives and publishing scores in hospital (n = 17); the prioritisation of feasible but high-impact pilots such as hand hygiene, starting small to show “the problem” and including monitoring, audit and feedback from the beginning of IPC implementation (n = 12); and putting focus on certain methods such as communication of positive audit and feedback culture (i.e. not punitive) and integration with national health monitoring and information systems (n = 6). Other unique ideas included the use of color-coded systems, assigning link nurses to lead monitoring, audit and feedback activities, conducting audits daily on intensive care units, monthly on selected wards, and every 3–6 months on the full hospital, integration with other monitoring tools for tuberculosis, patient safety, WASH, harmonization of monitoring indicators across states, use of phone applications (e.g. for hand hygiene observations), and linkage of monitoring indicators to accreditation, improvement plan or benchmarking (Additional file 1).

### Workload, staffing and bed occupancy

The seventh WHO IPC core component recommends that bed occupancy should not exceed the standard capacity of the facility and that staffing levels should be adequately assigned according to patient workload. Two frequent themes were identified as important for the implementation of this component (Table 1): the need for the participation of national level actors to set standards (e.g. nurse-patient ratio) and long-term advocacy with such actors (n = 11) and putting focus on certain methods such as the use of data and local research to set standards (n = 3). Other unique ideas included using link nurses, local solutions such as smaller beds in paediatric wards, decentralizing care when possible, task-sharing models, and use of outbreak data to increase political will for staffing and bed occupancy improvement (Additional file 1).

### Built environment, materials and equipment

The eighth WHO IPC core component recommends that patient care activities should be undertaken in a clean and/or hygienic environment, including all elements around WASH infrastructure and services and availability of appropriate IPC materials and equipment. The latter emphasises that materials and equipment to perform appropriate hand hygiene should be readily available at the point of care. Three frequent themes were identified as important for the implementation of this component (Table 1): the active involvement of IPC professionals in facility construction (n = 8); putting focus on certain elements of a multimodal strategy such as procurement of equipment for hand hygiene (n = 5); and promotion of long-term advocacy of WASH and integration with the health system (n = 3). Other unique ideas included the identification of evidence-based high-risk environmental points for process control in facility, starting with WASH training and assessment visits to wards and sterilization unit such as using the WHO WASHFIT tool, ensuring well-functioning incinerators, setting national WASH standards also for accreditation, the provision of training to Ministries overseeing facility construction, and regulation for IPC in facility construction (Additional file 1).

## Discussion

IPC experts from 27 countries across six WHO regions and two international organizations reported a range of IPC implementation experiences which could be categorized into frequent themes as well as unique implementation approaches for each of the WHO IPC core components.

These implementation themes can serve as useful qualitative evidence, especially considering that studies on the effectiveness of IPC implementation strategies in low-resource settings remain limited and rarely provide detailed explanations on how results were achieved and what were the success factors. A systematic review on IPC implementation strategies for nurses in Sub-Saharan Africa found that few implementation methods were reported, the majority reporting mainly didactic education and training approaches [15]. Another literature review on IPC measures for labour and delivery in developing countries only found two studies in upper-middle income countries which reported the impact of IPC strategies, whereas other studies focused mainly on challenges such as lack of laboratory services and surveillance data, poor design of building facilities and water and sanitation systems, insufficient numbers of beds and staff, and ill-defined manager roles [16–18]. Reviews on the evidence of IPC in the Asia–Pacific region have reported some observational and interventional studies in low-resource settings, particularly on the promotion of hand hygiene and antimicrobial stewardship, but these reviews have also highlighted important gaps, i.e. the lack of details on implementation aspects such as the organisation of IPC, leadership, types of education, and surveillance targets [19, 20].

Despite the need for improved evidence in low-resource settings, an increasing number of important studies in low-resource settings have been published on the impact of IPC multimodal strategies (WHO IPC core component 5), using before-after study designs [21–26]. The findings of our qualitative analysis on multimodal strategies align with the intervention components used in these impact evaluation studies, such as leadership buy-in, monitoring, audit and feedback, practical training, and promotion of safety culture. For example, a study by Phan et al. found significant sustained effects of a comprehensive multimodal hand hygiene campaign in Vietnam from 2010 to 2018 [27]. Hand hygiene compliance improved from 21.5% to 75.1% and HAI-incidence decreased from 1.10 to 0.45 episodes per 1000 patient-days respectively. The campaign intervention included alcohol-based hand rub (ABHR) production, annual trainings with focus group discussions, role-playing games, practical exercises and scientific lectures, patient educational videos, workplace poster reminders, hand hygiene competitions with modest prizes and “how-to-handrub”-related dance performances, audit and feedback, and signed commitment pledges by hospital leadership. Another study by Allegranzi et al. evaluated the impact of an intervention consisting of multiple surgical site infection (SSI) prevention measures and an adaptive approach to improve teamwork and the safety climate in five African hospitals from 2013 to 2015 [22]. They found that SSI incidence significantly decreased from 8.0% to 3.9% (p < 0.0001), albeit some heterogeneity between sites. Interviews with those who participated in the intervention study also identified influential individuals, peer-to-peer learning, infrastructure and momentum from previous projects and timely feedback as IPC facilitators [28]. In South Africa, a study by Richards et al. assessed a campaign to reduce central-line associated bloodstream infection (CLABSI) in 49 hospitals from 2010 to 2016 [23]. The campaign started with commitment from management as well as IPC doctors and nurses and monitoring of monthly infection rates and later included multidisciplinary regional learning sessions, benchmarking and audits. CLABSI rates decreased from a mean of 3.6 to 0.13 per 1000 central-line days (p < 0.001), respectively.

Selected international organizations have also published summary statements of expert opinions on IPC implementation examples in low-resource settings. For example, the *International Society for Infectious Diseases* published a position statement in 2020 on the implementation of surgical site infection surveillance in low- and middle-income countries [29]. This statement highlights implementation steps including identifying index operations for targeted surveillance, identifying IPC “champions” and empowering health care workers, using multimodal improvement measures, positioning hand hygiene programs as the basis for IPC initiatives, and use of telecommunication devices for surveillance and health outcome follow-ups. The findings of our qualitative analysis for HAI surveillance (WHO core component 4) corroborate this statement, highlighting the prioritisation of pilots such as surgical site infection, stepwise scale-up to electronic forms, multidisciplinary collaboration and mentorship, careful consideration of definitions, and “data for action.”

In our qualitative analysis, efforts were made to ensure the validity of identified themes by iteratively reviewing codes and verifying findings with multiple experts. However, this does not exclude the possible study limitation that the results may have been influenced by the researchers’ personal biases and views. The cut-off for defining “frequent implementation themes” (≥ 3 occurrences) was a subjective decision by the researchers, albeit informed by the distribution of responses. Additionally, the convenience sample of interviewees covered all WHO regions but the geographical breakdown may have also influenced the results (≥ 3 occurrences).

## Conclusion

The IPC implementation themes and unique approaches identified for each of the WHO IPC core components in this qualitative analysis bring significant added value to the body of evidence and real-life experiences supporting IPC implementation in low-resource settings. These themes and ideas were used to inform the development of WHO practical manuals for the implementation of the IPC core components [30]. They can act as a reference and provide inspiration for the development of effective IPC programmes as well as management of HAIs and infectious disease threats such as COVID-19.

## Supplementary Information

**Additional file 1: Table**. Other identified ideas (< 3 occurrences) concerning IPC implementation learned lessons in low-resource settings according to each WHO core component.

## Data Availability

The data (interview notes) are available from the corresponding author on reasonable request.

## Abbreviations

AMR: Antimicrobial resistance
CLABSI: Central-line associated bloodstream infection
COVID-19: Coronavirus disease-2019
HAIs: Health care-associated infections (HAIs)
INICC: International Nosocomial Infection Control Consortium
IPC: Infection prevention and control
IT: Information Technology
NHSN: National Healthcare Safety Network
SARS-CoV-2: Severe acute respiratory syndrome coronavirus 2
SSI: Surgical site infection
WASH: Water Sanitation and Hygiene
WHO: World Health Organization
