# Supplementary material for: Infection prevention and control (IPC) implementation in low-resource settings: a qualitative analysis
Source: Antimicrob Resist Infect Control. 2021 Jul 31;10:113. doi: 10.1186/s13756-021-00962-3 (PMC8325287; doi:10.1186/s13756-021-00962-3)
Supplement: Supplementary file 1 — Additional file 1: Table. Other identified ideas (< 3 occurrences) concerning IPC implementation learned lessons in low-resource settings according to each WHO core component. [file 13756_2021_962_MOESM1_ESM.docx]

**Additional file**

**Table. Other identified ideas (<3 occurrences) concerning IPC implementation learned lessons in low-resource settings according to each WHO core component**

| **Theme** | **Sub-themes** | **N*** |
| --- | --- | --- |
| **Core Component 1: IPC programme** | | |
| Use specific activities or opportunities as “catalysts” for advocacy (n=8) | Use research grant programmes | 2 |
|  | Use outbreaks to build political will | 2 |
|  | Patient associations can also help to promote the need for IPC | 2 |
|  | Build public awareness and leverage concerns (e.g. dirty hospitals) to promote IPC | 1 |
|  | Appeal to moral obligation | 1 |
| Promote linkages with health system (n=4) | Link IPC personnel and team with the nursing directorate | 1 |
|  | Link IPC personnel and team with the patient safety team | 1 |
|  | Create national cross-departmental committee and IPC-specific technical working groups | 1 |
|  | Consider role of political structures and collaboration needed, e.g. decentralized states | 1 |
| National IPC association can drive IPC improvement (n=4) | National IPC association can be active in hosting conferences | 2 |
|  | Some IPC activities were started in selected “model hospitals” and professionals from these facilities then created an IPC association | 2 |
| Need an approach to maintain “continuous” advocacy (n=2) | Send regular emails copying in senior leadership/managers | 2 |
| Use a stepwise approach to build required resources (n=2) | First asking the administration to dedicate one to two working days to IPC | 2 |
| May need normative actions to convince stakeholders (n=2) | International norms or standards should be cited | 2 |
| **Core Component 2: IPC guidelines** | | |
| Consider specific approaches to operationalize guidelines (n=10) | Create a small monthly budget for guideline implementation, e.g. shift from other areas | 2 |
|  | Link guidelines directly to standard operating procedures | 2 |
|  | Set implementation deadlines for hospitals | 2 |
|  | Link guidelines to short "how-to guides/survival kits” that personnel can carry on them | 2 |
|  | Link guidelines to research activities | 1 |
|  | Link guidelines to smartphone applications | 1 |
| May first need external technical assistance (n=2) | Selected professionals should first receive IPC training and they can then better assist with guideline development and adaption | 2 |
| **Core Component 3: IPC education and training** | | |
| Promote linkages with health system and sustainability (n=6) | Integrate competencies into job descriptions and performance reviews | 2 |
|  | Require participants to pay a small fee to sustain programme | 1 |
|  | Collaborate with other relevant Ministries to develop, deliver and maintain trainings | 1 |
|  | Collaborate with universities to develop, deliver and maintain trainings | 1 |
|  | Collaborate with national IPC association to develop, deliver and maintain trainings | 1 |
| Consider specific training methods (n=5) | Always include IPC in new employee orientation | 2 |
|  | Link training to mentorship initiatives | 1 |
|  | Also conduct trainings specific for administrators and managers | 1 |
|  | Use regional meetings to keep experts updated | 1 |
| Ensure certain training content elements (n=4) | Link training content to what is currently in guidelines or standard operating procedures | 2 |
|  | Start with the recognized subject of hand hygiene | 1 |
|  | Include communication skills in training content | 1 |
| Foster local IPC leadership during trainings (n=1) | Identify local champion trainers and trainees at the regional health services level | 1 |
| **Core Component 4: Health care-associated infection (HAI) surveillance** | | |
| Prioritise feasible but high-impact starting points or pilots (n=7) | Start with neonatal sepsis pilot | 2 |
|  | Consider mandatory surveillance, e.g. for multidrug-resistant organisms | 1 |
|  | Start with wound infection pilot | 1 |
|  | Start with clinical surveillance i.e. patient interviews, round observations, chart reviews | 1 |
|  | Could show tuberculosis data to show "problem" to advocate for HAI surveillance | 1 |
|  | Could start surveillance as part of a research project | 1 |
| Promote “data for action” (n=7) | Regularly discuss data and opportunities for action with leadership, i.e. meetings, emails | 2 |
|  | Send monthly or annual reports | 2 |
|  | Display HAI rates on ward notice boards | 1 |
|  | Regularly discuss data on grand rounds | 1 |
|  | Use outbreak experiences to can increase political will | 1 |
| Carefully consider definitions and data quality processes (n=5) | Prioritize first process indicators then outcome surveillance and risk stratification | 2 |
|  | Use International Nosocomial Infection Control Consortium (INICC) for benchmarking | 2 |
|  | Advocate for harmonized definitions/methods across states, i.e. strong communication | 1 |
| Ensure multidisciplinary collaboration, mentorship (n=3) | Advocate for leadership buy-in on the important need for surveillance | 2 |
|  | Collaborate with IT department and other public health services for surveillance | 1 |
| **Core Component 5: Multidmodal strategies for implementation of IPC interventions** | | |
| Put focus on certain elements of multimodal strategies (n=8) | Local production of alcohol-based hand rub is a key element | 2 |
|  | Integration of bundle interventions and quality checklists is a key element | 2 |
|  | Establishing facility goals and standards is a key element | 1 |
|  | Environmental cleaning is a key element | 1 |
|  | National committee sub-group on multimodal strategies is a key element | 1 |
|  | Collaboration with private and academic institutions is a key element | 1 |
| Promote activities to clearly communicate and advocate for multimodal strategies (n=6) | Outbreaks can show the important of multimodal strategies and enable political will | 2 |
|  | Use surveillance data to demonstrate need and impact of multimodal strategies | 2 |
|  | Develop multimodal strategies in the context of a research study | 1 |
|  | Use the support to prevent and control AMR to advocate for multimodal strategies | 1 |
| Prioritise feasible but high-impact starting points or pilots (n=5) | Focus where “it hurts the most”, e.g. outbreaks | 2 |
|  | Start with health care-associated pneumonia or tuberculosis pilot | 1 |
|  | Prioritise low-cost interventions to promote sustainability | 1 |
|  | Adapt successful multimodal strategy examples from other countries | 1 |
| **Core Component 6: Monitoring/audit of IPC practices and feedback** | | |
| Put focus on certain methods (n=13) | Use color-coded monitoring and feedback system to improve understanding of results | 2 |
|  | Use link nurses for implementation for monitoring/audit and feedback activities | 2 |
|  | Conduct audits on rounds: Intensive care (daily), selected ward (monthly), full hospital (every 3-6 months) | 2 |
|  | Integrate IPC monitoring and feedback with tools used for tuberculosis | 2 |
|  | Integrate IPC monitoring and feedback with patient safety tools | 1 |
|  | Integrate IPC monitoring and feedback with WASH tools | 1 |
|  | Harmonize monitoring indicators across states | 1 |
|  | Use external surveyors from regional health services | 1 |
|  | Create phone application for monitoring and feedback, e.g. hand hygiene observations | 1 |
| Prioritise feasible but high-impact starting points or pilots (n=7) | Start with device-associated infections, e.g. urinary or bloodstream, process indicators | 2 |
|  | Adapt international monitoring and audit tools | 2 |
|  | Can start with manual data collection, e.g. round observations, and scale up to electronic | 2 |
|  | Start with outbreak data to increase political will for change | 1 |
| Promote “data for action” (n=3) | Link monitoring indicators and feedback to accreditation steps | 2 |
|  | Link monitoring indicators and feedback to improvement plan | 1 |
|  | Develop benchmarking system and show progress to leadership, e.g. facility-based, INICC | 1 |
| **Core Component 7: Workload, staffing and bed occupancy** | | |
| Put focus on certain methods (n=7) | Use outbreak data to increase political will for staffing and bed occupancy improvement | 2 |
|  | Use link nurses to improve workload and staffing | 2 |
|  | Consider adaptions to meet bed occupancy standards, e.g. smaller beds in paediatric wards, decentralize care when possible | 1 |
|  | Advocate for increased health care worker salaries | 1 |
|  | Use task-sharing models to improve workload and staffing | 1 |
| **Core Component 8: Built environment, materials and equipment for IPC** | | |
| Put focus on certain elements of a multimodal strategy (n=9) | Identify evidence-based high-risk environmental points for process control in facility | 2 |
|  | Start with WASH education, training, and visits to wards and sterilization unit | 2 |
|  | Start with ensuring well-functioning incinerators | 2 |
|  | Set WASH standards at the national level | 1 |
|  | Start with waste management pilot, e.g. coloured bins | 1 |
|  | Conduct monitoring and feedback using WASHFIT developed by WHO | 1 |
| Promote long-term advocacy and integration with health system (n=5) | Promote IPC culture early in school WASH and hand hygiene projects | 2 |
|  | Link IPC with national committee workgroup on infrastructure and/or WASH | 1 |
|  | Set WASH standards for healthcare facility accreditation | 1 |
|  | Develop sustainable strategy for local production of alcohol-based hand rub and (preoperative) antiseptic skin solution | 1 |
| IPC professionals should be actively involved in facility construction (n=4) | Provide IPC training to Ministry or governmental body overseeing facility construction | 2 |
|  | Develop regulation to enforce involvement of IPC professionals in facility construction | 2 |

*Themes and sub-themes are listed in order of decreasing frequency for each WHO core component of IPC programmes;

**Acronyms: AMR (Antimicrobial Resistance), HAI (Health care-Associated Infections), INICC (International Nosocomial Infection Control Consortium), IPC (Infection Prevention and Control), IT (Information Technology), WASH (Water Sanitation and Hygiene), WASHFIT (Water and sanitation for health facility improvement tool), WHO (World Health Organization)
